# p16INK4A expression is frequently increased in periorbital and ocular squamous lesions

**DOI:** 10.1186/s13000-015-0396-8

**Published:** 2015-09-24

**Authors:** Peter J. Kobalka, Jean-Paul Abboud, Xiaoyan Liao, Karra Jones, Bradford W. Lee, Bobby S. Korn, Don O. Kikkawa, Jonathan H. Lin

**Affiliations:** Department of Pathology, University of California San Diego and VA San Diego Healthcare System, San Diego, CA USA; Shiley Eye Center and Department of Ophthalmology, University of California San Diego and VA San Diego Healthcare System, San Diego, CA USA; Department of Pathology, School of Medicine, UC San Diego, 9500 Gilman Dr., La Jolla, CA 92093-0612 USA

## Abstract

**Background:**

p16 expression is a well established biomarker of cervical dysplasia and carcinoma arising from high risk human papilloma virus infection. Increased p16 expression is also seen in squamous neoplasms arising at other sites, including head, neck, and oropharyngeal tract. Squamous lesions are also frequently encountered at ocular surface and peri-orbital skin sites, but the prevalence of increased p16 expression in these lesions has been poorly studied.

**Methods:**

We retrospectively surveyed 13 ocular surface and 16 orbital squamous lesions biopsied at UC San Diego Healthcare System and VA San Diego Healthcare System for p16 expression by immunohistochemistry. These cases included ocular surface lesions with diagnoses of conjunctival intraepithelial neoplasm (CIN) and squamous cell carcinoma in situ. Peri-orbital eyelid biopsies included lesions with diagnoses of SCCis and invasive squamous cell carcinoma. We performed multivariate logistic regression, followed by student’s T-test or Fisher's exact test to determine if there were statistically significant associations between p16 immunoreactivity and patient age, gender, diagnosis, and ethnicity. Statistical significance was defined as p < 0.05.

**Results:**

We found an unexpectedly large prevalence of strong nuclear and cytoplasmic p16 immunoreactivity in our cases. Almost all of the ocular surface squamous lesions were diffusely positive for p16 expression (12/13). All of the periorbital lesions showed diffuse p16 immunoreactivity (16/16). Altogether, 28/29 lesions tested showed strong and diffuse p16 expression. We found no statistically significant correlation between p16 expression and patient age, gender, ethnicity, or diagnosis. In 6 of the peri-orbital biopsies, we had sufficient tissue to assess high-risk HPV expression by in situ hybridization. Interestingly, all of these cases were negative for HPV, despite strong p16 expression.

**Conclusion:**

Strong p16 expression was observed in virtually all of the ocular surface and peri-orbital squamous neoplasms in our study. The relationship between p16 expression and HPV infection in ocular surface and peri-orbital sites requires further investigation.

## Background

HPV is linked to development of cervical, anogenital, head/neck, and lung squamous lesions and carcinomas [1–5]. Increased p16^INK4A^ expression is seen in HPV-associated squamous lesions, and the strength and subcellular staining pattern of p16 immunohistochemical reactivity correlates closely with the degree of dysplasia, with higher-grade lesions showing intense, diffusely positive nuclear and cytoplasmic p16 expression in affected squamous cells [6–14]. Increased p16^INK4A^ expression arises from neutralization of cellular p53 and pRb tumor suppressor proteins by HPV E6 and E7 oncogenes in infected cells [15, 16]. Low-risk HPV subtypes typically show less p16^INK4A^ induction because they produce E6 and E7 viral oncogenes with lower ability to neutralize endogenous tumor suppressors [17]. Therefore, p16^INK4A^ expression is commonly employed as a surrogate marker of infection by high-risk HPV subtypes.

Malignant squamous lesions of ocular and peri-ocular structures include conjunctival intraepithelial neoplasia (CIN) to frank invasive squamous cell carcinoma (SCC). HPV has also been detected in prior studies of ocular and peri-ocular squamous lesions and suggested to contribute to the pathogenesis of squamous lesions in the eye [18–21]. However, very few studies have examined p16 expression in squamous lesions resected from the eye, and there have been conflicting findings from these reports. In a cohort of HIV seropositive patients in Africa, Mwololo and colleagues findings found strong p16 immunoreactivity in 67 % (39 of 58) of SCC conjunctival biopsies [22]. However, in a study of ocular surface and conjunctival squamous lesions performed in Australia, Woods and colleagues found p16 immunoreactivity in only 6.5 % (3 of 46) ocular surface squamous neoplasia biopsies and 12.5 % (3 of 24) conjunctival SCCs [23]. Interestingly, p16 immunoreactivity completely correlated with HPV detection by PCR in this study [23].

In our study, we hypothesized that p16 immunoreactivity was significantly increased in malignant ocular squamous lesions. To examine this, we examined 29 ocular surface and periorbital squamous lesions excised at our institution that comprised malignant (CIN and SCC) ocular squamous neoplasms. We performed p16 IHC analysis on all lesions, followed by HPV ISH on a subset of p16-positive cases. We compared the histopathologic and molecular findings with clinical data to identify potential significant correlations between p16 expression in ocular squamous lesions and clinical characteristics of our patient population.

## Methods

### Tissue specimens

We examined a total of 29 ocular surface and periorbital eyelid squamous lesion cases retrieved from the VA San Diego and UCSD Medical Center between July 2010-May 2014. These cases were routinely fixed in 10 % formalin and embedded in paraffin. H&E (hematoxylin and eosin)-stained slides were reviewed and classified by an ophthalmologic pathologist. All lesions with a diagnosis of CIN or SCC (in situ or invasive) were included in this study. The study was approved by the Institutional Review Boards at UCSD and VA San Diego.

### p16 immunohistochemistry

Immunohistochemical studies for p16 were performed using a Ventana Benchmark Ultra. The p16 antibody used was a monoclonal mouse antibody (clone JC8, Santa Cruz Biotechnology; dilution 1:80). Lesions were considered positive for p16 when both cytoplasmic and nuclear staining was observed in epithelial cells. Staining in dendritic cells and non-epithelial cells was disregarded.

### HPV *in-situ* hybridization

*In-situ* hybridization was performed on formalin-fixed, paraffin-embedded tissue by ARUP laboratories (Salt Lake City, Utah). The test was run on a Ventana Bentana Benchmark Ultra (Ventana Medical Systems, Inc), using the INFORM HPV II FAMILY 6 (ASR) probe. A probe set for high-risk HPV [16, 18] were employed (catalog #2002899). This test is CLIA-certified for clinical use and widely performed for evaluation of high risk HPVs in suspicious tissue biopsies.

### Statistical analysis

Statistical analysis was performed on CINs and SCCs. The correlation between p16 immunoreactivity and patient’s age, gender, diagnosis, and ethnicity was first analyzed by Multivariate Logistic Regression using SAS 9.3, followed by individual student’s T-test (for age difference) or Fisher’s exact test (for gender, ethnicity, or diagnosis differences). A difference was considered statistical significant if p < 0.05.

## Results

### p16 expression by immunohistochemistry

**Fig. 1:**
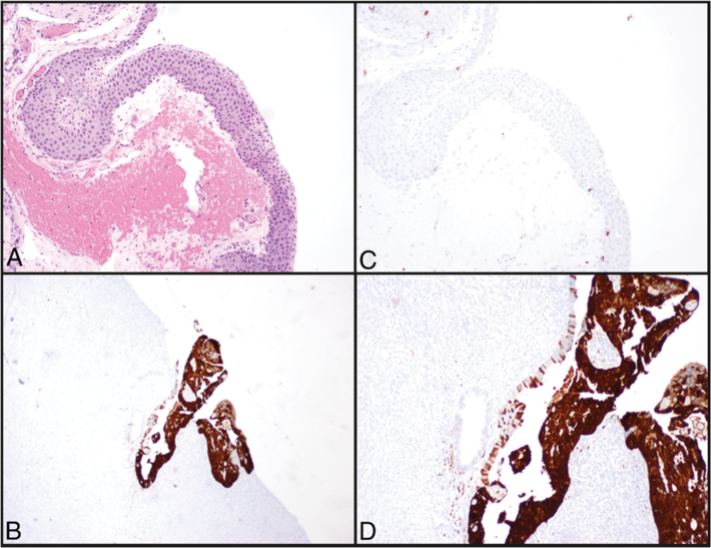
**a** Histology of the negative control, taken from a pterygia (20x, magnification). **c** Immunohistochemical staining for p16 in the same case (20x magnification). Note the lack of staining in epithelial cells. **b**, **d** Positive control p16 immunohistochemical staining, taken from a squamous cell carcinoma in-situ from cervix (4x and 20x, respectively). Note both cytoplasmic and nuclear reactivity

**Fig. 2:**
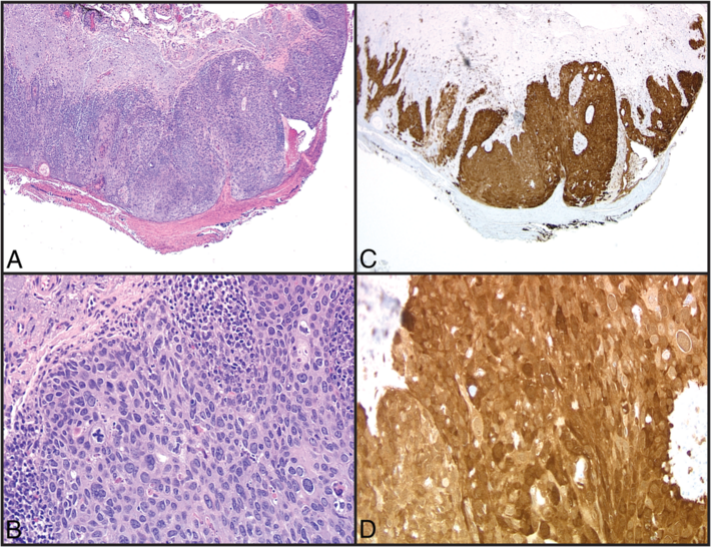
**a**, **b** Histology of a squamous cell carcinoma in situ from eyelid biopsy (4x and 20x, respectively). **c**, **d** Immunohistochemical staining for p16 in the same case (4x and 20x, respectively). Note the extensive cytoplasmic and nuclear reactivity

**Fig. 3:**
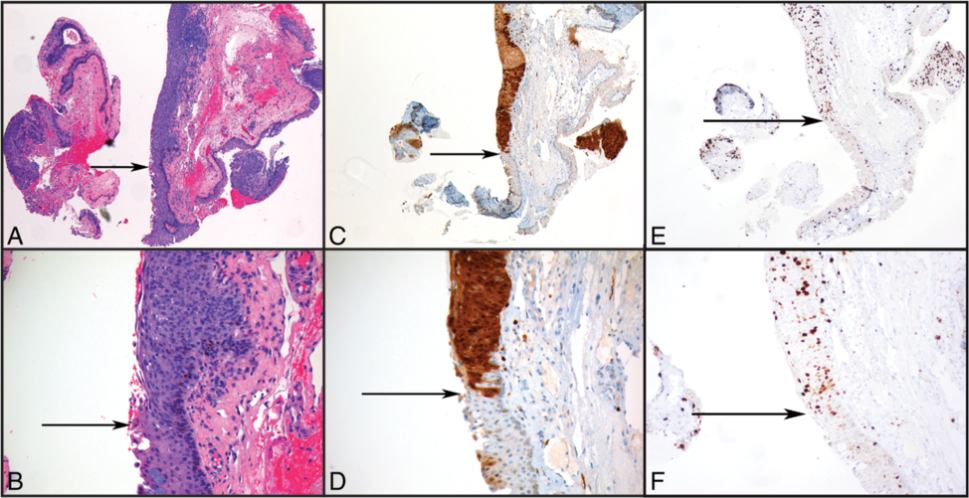
**a**, **b** Histology of an in-situ squamous cell carcinoma of the conjunctiva from a patient with HIV (4x and 20x, respectively). **c**, **d** Immunohistochemical staining for p16 in the same case (4x and 20x, respectively). Note the lack of p16 staining in adjacent normal conjunctiva with goblet cells. Arrows mark the transition between normal and dysplastic epithelium. **e**, **f** Immunohistochemical staining for Ki-67 (MIB-1). Note full-thickness reactivity in dysplastic epithelium

**Fig. 4:**
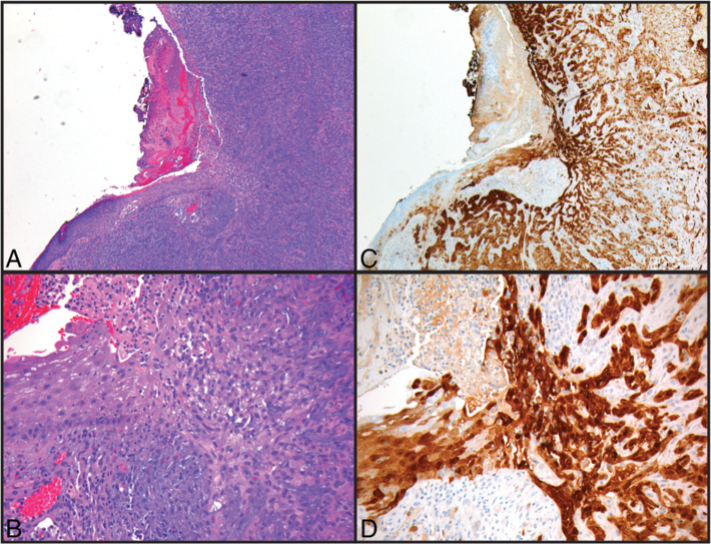
**a**, **b** Histology of an invasive squamous cell carcinoma (4x and 20x, respectively). **c**, **d** Immunohistochemical staining for p16 in the same case (4x and 20x, respectively). Note both cytoplasmic and nuclear reactivity

A total of 29 cases were evaluated for p16 immunoreactivity (Table 1). Patients ranged in age from 37 to 88 and included 11 women and 18 men with White ethnicity (by self-report) being the most common. The majority of patients were seronegative for HIV (27/29). Negative p16 immunoreactivity was defined as lacking cytoplasmic and nuclear labeling of the squamous keratinocytes (Fig. 1), while p16 was considered positive when there was both strong cytoplasmic and nuclear labeling of dysplastic keratinocytes in the lesion. By this criteria, 28 of the 29 cases showed strong p16 immunoreactivity of dysplastic squamous cells (Figs. 2, 3, and 4 and Table 1). When grouped by diagnosis, all of the SCCs (20/20) were p16-positive (Figs. 2 and 4, Table 1), and 8/9 CINs were p16-positive (Table 1).

**Table 1:** Patient clinico-pathologic profiles

Age	Sex	Anatomic site	Ethnicity	HIV status	Pathologic diagnosis	P16 immunoreactivity	P16 IHC staining pattern	ISH
71	M	Eyelid	Unknown	Negative	Invasive SCC	Positive	Nuc and cyto	^a^
85	M	Eyelid	White	Negative	Invasive SCC	Positive	Nuc and cyto	^a^
59	M	Eyelid	White	Negative	SCC in situ	Positive	Nuc and cyto	^a^
46	M	Conjunctiva	White	Negative	invasive SCC	Positive	Nuc and cyto	^a^
78	F	Eyelid	White	Negative	SCC in situ	Positive	Nuc and cyto	^a^
37	M	Conjunctiva	Hispanic	Negative	SCC in situ	Positive	Nuc and cyto	Negative
88	F	Eyelid	White	Negative	SCC in situ	Positive	Diffuse nuc and cyto	Negative
89	F	Eyelid	White	Negative	SCC in situ	Positive	Diffuse nuc and cyto	^a^
67	F	Eyelid	White	Negative	Invasive SCC	Positive	Diffuse nuc and cyto	^a^
77	M	Conjunctiva	White	Positive	CIN	Positive	Diffuse nuc and cyto	^a^
76	F	Eyelid	White	Negative	SCC in situ	Positive	Diffuse nuc and cyto	Negative
47	M	Cornea	White	Negative	SCC in situ	Positive	Diffuse nuc and cyto	^a^
45	F	Eyelid	White	Negative	Invasive SCC	Positive	Diffuse nuc and cyto	Negative
76	F	Eyelid	White	Negative	SCC in situ	Positive	Scattered nuc and cyto	^a^
61	M	Eyelid	White	Negative	Invasive SCC	Positive	Focal nuc and cyto	Negative
88	F	Eyelid	White	Negative	Invasive SCC	Positive	Diffuse nuc and cyto	Negative
88	F	Eyelid	White	Negative	SCC in situ	Positive	Patchy nuc and cyto	^a^
88	F	Eyelid	White	Negative	SCC in situ	Positive	Focal nuc and cyto	^a^
45	M	Eyelid	White	Negative	SCC in situ	Positive	Diffuse nuc and cyto	^a^
65	M	Eyelid	Unknown	Negative	SCC in situ	Positive	Diffuse nuc and cyto	^a^
76	M	Conjunctiva	Hispanic	Negative	CIN	Positive	Focal nuc and cyto	^a^
32	F	Conjunctiva	White	Negative	CIN	Positive	Scattered nuc and cyto	^a^
56	M	Conjunctiva	Hispanic	Positive	CIN	Positive	Scattered nuc and cyto	^a^
46	M	Cornea	White	Negative	CIN	Positive	Diffuse nuc and cyto	^a^
64	M	Cornea/conjunctiva	Unknown	Negative	SCC in situ	Positive	Diffuse nuc and cyto	^a^
72	M	Conjunctiva	White	Negative	CIN	Positive	Diffuse nuc and cyto	^a^
56	M	Conjunctiva	White	Negative	CIN	Positive	Scattered nuc and cyto	^a^
40	M	Conjunctiva	Hispanic	Positive	CIN	Negative	N/A	^a^
70	M	Conjunctiva	White	Negative	CIN	Positive	Diffuse nuc and cyto	^a^

*Nuc* Nuclear, *Cyto* cytoplasmic

^a^Not performed

### HPV detection by *in-situ* hybridization

HPV *in-situ* hybridization against HPV genotypes 6, 11, 16, and 18 was performed on 6 lesions in total that were intensely and diffusely positive for p16 by immunohistochemistry. Of six squamous cell carcinomas tested, none were positive for HPV. CIN cases had insufficient remaining tissue in the blocks to perform the HPV ISH assay due to the small size of the conjunctival biopsy.

### Clinical and statistical correlation

We next examined if positive p16 expression correlated with pathologic type of squamous lesion, patient age (less than or older than 50), patient gender, and ethnicity (White, Hispanic) by multivariate logistic regression, Student’s t-test, and Fischer’s exact test. No significant differences were seen between the pathologic types of lesions tested and p16 positivity (p >0.05). In addition, there were no significant differences between p16 positivity and age, gender, ethnicity or diagnosis.

## Discussion

Our study reveals a surprisingly high prevalence of p16 immunoreactivity in ocular squamous lesions from our patient population, but interestingly, these squamous lesions with strong p16 expression did not show HPV 6, 11, 16, and 18 genetic material by ISH. We discuss some potential causes for the increased p16 expression but low HPV infection observed in our study.

P16 immunoreactivity is well established as a biomarker for HPV infection in sites such as cervix, and the vast majority of HPV-associated cancers show increased p16 expression [12–14, 24, 25]. However, p16 induction has also been observed after inflammatory conditions, after total body irradiation, and with prolonged genomic damage, independent of HPV infection [26, 27] and in a recent study of bladder SCC, about 1/3 of SCC cases showed p16 immunoreactivity but no HPV genetic material by in situ hybridization [28]. Similarly, increased p16 expression did not correlate with HPV infection in SCC from lung, skin and esophagus [29]. In our ocular squamous neoplasms, it is possible that increased p16 expression could arise from stresses other than HPV infection, and indeed, solar damage was previously proposed as a risk factor for increased HPV infection in eye disease [19].

Our HPV ISH test was performed by ARUP labs, using a commercially available limited HPV ISH probe against HPV genotypes 6, 11, 16, and 18 that is widely used to identify HPV infection in human tissue specimens. These HPV genotypes are widely observed in cervical squamous lesions but their trophism to other squamous sites is less clear. Ateenyi-Agaba and colleagues isolated several uncharacterized HPV genotypes from their conjunctival SCC lesions [21]. It remains possible that in our specimens, other HPV genotypes could be present and account for the increased p16 expression. A broader spectrum probe for more HPV genotypes could help reconcile p16 positive lesions that were initially negative [29].

HPV detection techniques vary in their sensitivity [30]. Our study employed a CLIA-approved ISH assay commonly used for cervical biopsies at our institution as a confirmatory test for HPV in p16 positive ocular squamous lesions. RT-PCR testing is more sensitive for HPV testing [31, 32], and, in one study of oropharyngeal SCCs, many ISH negative SCCs of the oropharynx were positive by PCR [33]. Repeat HPV testing by different methodologies may be required to identify HPV in cases with high clinical suspicion. Further follow-up studies using more sensitive and broader methodologies may be helpful to determine if p16 expression in ocular squamous lesions corresponds to HPV infection.

## Conclusion

Our studies showed that p16 expression is frequently increased in malignant squamous lesions arising at the conjunctiva or adjoining eyelid.
